# The London Hospital and the "Pall Mall Gazette"

**Published:** 1893-08-05

**Authors:** 


### The Institutional Workshop.

### THE LONDON HOSPITAL AND THE "PALL MALL GAZETTE."

(By Our Special Commissioner.)

We have already expressed our opinion of the character of the articles which have been published in the *Pall Mall Gazette*, and have shown that the statement that they were written in consequence of the appeal made in our articles published in The Hospital of June 3rd and 10th, 1893, is not the statement of truth. We purpose to-day to analyse the articles, and to bring out (*a*) the statements that are false; (*b*) the statements that are true; and (*c*) the statements that are misleading, *i.e.*, those that are neither false nor true. In order that we might secure accuracy we have, in addition to a personal inspection, caused each item in the *Pall Mall's* indictment to be carefully checked, and have tested it in more than one way. What follows may therefore be taken as a plain unvarnished tale, containing absolute facts, and we do not think that there is any fair-minded reader who will not feel—as we said last week—that the series of articles which have appeared in the *Pall Mall Gazette* on the London Hospital are among the most disgraceful, wanton, and aggravated libels which have ever appeared in an English paper.

(*a*) Statements that are False.

(1) That the *Pall Mall Gazette's* Special Commissioner, having read the appeal in The Hospital of June 3rd, resolved to enter the London Hospital as a nurse; did so enter, and that what she states in the articles was the result of her experience as a paying probationer during three months' residence.

This statement is proved to be untrue, because no paying probationer was admitted into the London Hospital between June 3rd and the date when the first article appeared in the *Pall Mall*, with the exception of a lady who entered on July 3rd, who is still a probationer in the hospital, and who is certainly not the writer of these articles.

(2) "That the waste of good food in the wards is simply shocking."

This statement is demonstrably false, as anyone may ascertain who will take the trouble to visit the hospital and test the statement on the spot. The commissariat of a hospital is a complicated business, and the administration has no alternative but to supply daily to each patient whatever food, *i e.*, diets, the medical attendant may order. These diets are sent up from the kitchens, and are served out with care and exactitude. As a rule, the diets are eaten, but the amount of food consumed in a ward must vary from day to day, according to the state of health of each patient it contains. When, as at the London Hospital, surplus and uneaten bread is collected and utilized in puddings, or sold to a contractor, and when the contents of the swill-tub—the only safe receptacle for all other kinds of broken food—are also sold, the charge of waste must stand condemned as false and unfounded. It must necessarily happen in a large establishment, whatever be the care exercised in the kitchen and store departments, that a bad egg may find its way into a custard pudding, that milk may turn sour, and that even beef-tea, however carefully prepared, may occasionally be found unpalatable. Hence there is no substance in the accusation that a custard pudding, or quantities of milk, or even beef-tea, have been thrown away on particular days in a particular ward. Again, having regard to the danger of infection which must attach to absorbent materials which have been exposed to the atmosphere of a crowded ward in a clinical hospital, the suggestion that the broken victuals, soup, and beef-tea which the patients have been too ill to eat, should be given to "the poor people outside the hospital" is a patent absurdity, which clearly proves the writer's ignorance of the feelings of the poor, of what is due to them, and of the dangers which attend the treatment of all classes of disease in a great hospital under the most favourable conditions.

The *Pall Mall Gazette's* correspondent states: "I cannot sing the praises of this kind of management, and be truthful at the same time." It is not so much a matter of truthfulness as a matter of common sense, knowledge, and sweet reasonableness. Certainly this correspondent displays little common sense, less knowledge, and no sweet reasonableness whatever. Of course the waste food is not good in the sense that it is not suitable for healthy people to eat, so that it would be an insult to offer it to the poor. We feel, however, it would be a just punishment to feed the *Pall Mall Gazette's* correspondent upon it for a month or six weeks, in order to demonstrate to the editor and readers of that paper how dangerous such a diet would in practice prove.

(3) That there are too few nurses as compared with the number of patients.

It is not true that there are altogether too few nurses, if any regard is to be had to the cost of maintaining a hospital bed. The fact is (see The Hospital, June 10th, page 175) there are more nurses to patients at the London Hospital than at any other hospital in the metropolis. The number of nurses to patients is considerably over one to three, whereas no other hospital has probably more than one nurse to every three patients, and the majority have less. During the take-in there must necessarily be great pressure upon the nurses in particular wards, but such pressure is only temporary, and there is no occupation in life where the worker does not have to face over-work and excessive pressure during brief periods of exceptional activity, like the take-in at a hospital.

(4) "The chief duty of the sisters is to act as a species of peripatetic inkstand in attendance on the doctors; their minor duties are occasional supervision of the nurses, carving the meat for the patients, and reporting tittle-tattle to the matron."

This monstrous statement is absolutely incorrect. The best sisters work harder than any of their nurses at actual nursing, and in addition exercise a most complete supervision over the whole of the wards committed to their care. Of course, some tittle-tattle there always must be in a large institution, but the *Pall Mall's* correspondent shows by the account of her own experience that the sisters, in fact, discourage everything of this kind, and impress upon nurses that "they are expected to do their work, and not to discuss matters belonging to departments other than their own."

That every opportunity is taken to give the Sisters at the London Hospital a thorough training is proved by the charge that the Home sister "during the time" the *Pall Mall Gazette's* Commissioner "was at the hospital was unfrocked, put into probationer's uniform, and sent back to finish her term in the wards." No doubt this Sister possessed the administrative gift, and, as occasion offered, she had been gradually trained in each branch of hospital work so as to make her technically efficient. This view is borne out by the knowledge that when a probationer is selected to do duty as a sister she is usually called Probationer-Sister, and put in a ward with the regular Sister for a time before taking sole charge of a floor.

(5) That the nurses on the private staff of the London Hospital are sweated to the extent of three-fourths of their earnings.

Not so. The facts are very simple, and are not peculiar to the London Hospital. Private nurses are of two kinds: (1) Those who prefer safety to adventure, and (2) those who prefer independence to safety. The latter class (2) become private nurses on their own account, which necessitates their taking the risk of finding patients to nurse and doctors to recommend them, and entails periods of idleness, when their limited savings are gradually diminished, and they have to suffer intense anxiety of mind. Such risks are a high price to pay for independence, and so a large number of nurses who apply themselves to private nursing prefer to join the private staff of an institution like the London Hospital. Here they receive from £30 to £40 a year in wages, with uniform, and are provided with a home when not at work, with food, and every other requisite, including medical attendance and care when ill. Thus, although a private nurse in full work in London to-day may earn a gross sum of from £80 to £100 a-year—a provincial nurse cannot earn nearly so much—whilst it may only cost a London nurse £40 or £50 to provide for her needs, her net income is often at best from £40 to £60 a year, but in reality it is frequently much less, as she is seldom in full work throughout an entire year, and, in case of illness, the lot of the private nurse is far from enviable. The institution nurse, on the other hand, is always sure of her £30 or £40 a year; she has few or no anxieties, because when she is ill or incapacitated by age the institution provides for her. Hence, to say the least, it is an injustice to the private nurse and to the institutions alike to talk of "sweating" under such circumstances, without any qualification, as the Commissioner of the *Pall Mall Gazette* does. It is only another proof that "fools rush in where angels fear to tread."

(6) That there is no direct supervision or forethought in the matter of the distribution of nurses at the London Hospital.

This is demonstrably untrue, seeing that the books kept at the London Hospital prove the exact contrary to be the case. The work of each member of the nursing staff is most carefully thought out; the amount of work to be done in each ward each day is taken into consideration; and the nurses are re-distributed from time to time so as to provide that the heaviest wards have an adequate number of nurses, and that the light wards are not over-manned. We may here further dispose of the statement that special nurses cannot be obtained for urgent cases by declaring, on the authority of the medical staff, that it practically never happens that an urgent demand for an extra nurse is not met. Again, the statement that the Matron never visits the wards is untrue. It is impossible for the Matron to visit every ward each day, for if she did she would have no time for anything else. So the Committee have appointed a deputy, who devotes her energies to this work and reports systematically to the Matron, who also visits the wards herself from time to time.

(7) That the milk is placed in open mugs on the lockers, and, not being covered, soon gets tainted from the unhealthy atmosphere of the wards.

This is not the fact. All milk not immediately required by the patients is kept in an ice-chest, with which every ward is provided, and the Sisters and nurses fetch it for the patients in small quantities, as they may require it. There is also a ludicrous exaggeration about this statement which the medical expert will not fail to appreciate.

(8) That the condition of cleanliness in which the "great unwashed" are kept is not above reproach, because it is not possible to bestow sufficient attention and care upon the patients.

This is absolutely untrue, and constitutes a malicious libel upon the Sisters and nurses and the Managers of the London Hospital. Had the editor of the *Pall Mall Gazette* gone down to the hospital and examined the patients for himself, he never would have permitted so scandalous a statement to appear in his columns.

(*b*) Statements that are True.

(1) That it is easier to procure admission as a paying than as a regular probationer.

We have no doubt that it is a mistake for any hospital to admit paying probationers for limited periods under different regulations from those which are enforced in the case of the regular probationer. Hospitals are places where serious business has to be transacted in a business-like way. To admit as a paying probationer the class of woman who is discontented at home, restless, sentimental, or impatient of discipline, as very many of those who apply for admission as paying probationers undoubtedly are, is to court similar attacks to those we are now analysing. We are strongly of opinion that the London and all well-administered hospitals would be well advised to close their doors once and for all against evils and annoyances of this description.

(2) That the Sister who is in charge of the Blizard Erysipelas Ward is also the Sister for the Isolation Ward, where there are from time to time cases of scarlet fever, measles, diphtheria, &c.

This is an undoubtedly wrong system, for which the medical staff must be held responsible, and no one else. They, and they alone, are the best judges of the risks of infection entailed by an arrangement of this kind, and, until they forbid it, the Committee and their officers are powerless to alter the system, which stands condemned on the face of it, and will, we hope, be corrected without loss of time.

(*c*) Statements that are Misleading.

(1) That probationers, who are only half-trained apprentices, are deprived of their regular training, and sent out to nurse private patients, as if they were thoroughly-trained nurses.

The chairman at the Quarterly Court, held in March last, declared this statement to be untrue, as it undoubtedly is in substance and in fact. We have classified it as misleading, because in a few cases a nurse of one year's standing has been sent to a private case as a part of her training to enable her to gain an insight into private nursing. It is fairly open to debate whether it would not be better to delay sending a nurse to a private case until she has seen two years' hospital work; at the same time no one can object to have a nurse of one year's standing to attend to them providing she has had the experience and training necessary to enable her to intelligently manage a particular case of illness, and that she has nursed a number of similar cases during her year of residence in the hospital.

(2) That some of the probationers are smiled upon, and converted into sisters before their term of apprenticeship has been served, over the heads of the staff-nurses, who have been several years at the hospital.

The work of a sister at the London, like that at St. Bartholomew's Hospital, is peculiar in the sense that it more nearly approaches that of a matron of a small hospital than that of a head nurse in charge of a large ward in a modern pavilion hospital. The London Hospital is so constructed as to necessitate an entire floor containing several wards, with fifty beds or upwards, being placed in charge of one sister, whose duties are largely administrative, the nursing being undertaken by a large staff of fourteen or more nurses under the sister's direction. A woman may be an excellent nurse, but be wholly incapable of administering a small hospital of this kind. Experience proves that the ablest sister need not necessarily be a certificated nurse, although it is essential that she should have a knowledge of the routine and work of the particular hospital where she is employed. No doubt as trained nursing extends, it will be possible, as it is certainly desirable to secure a sufficient number of certificated nurses who possess the gift of administration. In these circumstances, it is no hardship for a charge nurse to remain in that position, but quite the contrary, if, as frequently happens, the charge nurse does not possess the gifts which would enable her to discharge the administrative duties which fall upon the sister.

### A Brief General Summary.

We have now analysed the articles as they have appeared. It is neither our intention nor our wish to abrogate the functions of those whose privilege it is and will be to defend the London Hospital. We have merely endeavoured to fairly state the actual facts. The commissioner of the *Pall Mall Gazette* shows obvious bias all through. Such a statement as that, for instance, which relates to the serving of butter, tea, &c., and the description of it as a defect in the management of the London Hospital, when, on the whole, it adds materially to the comfort of the nurses, as they themselves admit, is a case in point. It is evidently malicious, and merely a piece of cheap sensational writing. Again the statement as to the washing of the dishes and plates by the probationers when it is done by the wardmaids, and when there are no dishes in the ordinary sense at the London Hospital, points in the same direction. The "ill and starving nurses," as described in the *Pall Mall Gazette*, have no existence, except in the imagination of this lady correspondent, and the charges as to the food supplied to the nurses are disproved and exposed by the diet-book, which shows what was actually served not only to the nurses, but to the sisters, matron's assistants, and every nursing mess throughout the institution. This book has been kept for a long time, and, had the writer known of its existence, she would surely never have published such direct evidence of her own unreliability and ignorance.

The article concludes with a paragraph entitled "What to Do." Here again the writer's ignorance of the facts is exposed to her own confusion. She gives eight classes of improvements, which seem to her to be absolutely necessary. Of these Nos. 1, 2, 3, 4, 5, and 8 are already enforced by the system under which the London Hospital is at present conducted. There has been continuous improvement and development every year for years past, and no man of business can fail to recognise the impossibility of introducing radical changes into a large establishment all at once. Of the remaining suggestions, No. 6 we have shown to be founded upon an ignorance of the true facts, whilst a knowledge of the circumstances involved in the adoption of the seventh suggestion must lead all reasonable people to agree, that on the whole, the balance of evidence favours the continuance of the present system. As to the suggestion that a ladies' committee should be appointed, we will only say that on the whole the best managed hospitals are those where there are no ladies' committees.

